# Supplementary material for: Bioactivity of Cooked Standard and Enriched Whole Eggs from White Leghorn and Rhode Island Red in Exhibiting In-Vitro Antioxidant and ACE-Inhibitory Effects
Source: Nutrients. 2021 Nov 25;13(12):4232. doi: 10.3390/nu13124232 (PMC8705232; doi:10.3390/nu13124232)

Supplementary Material

Bioactivity of Cooked Standard and Enriched Whole Eggs from White Leghorn and Rhode Island Red in Exhibiting In-Vitro Antioxidant and ACE-Inhibitory Effects

Emerson Nolasco [1], Mike Naldrett [2], Sophie Alvarez [2], Philip E. Johnson [1] and Kaustav Majumder [1,*]

[1] Department of Food Science and Technology, University of Nebraska-Lincoln, Lincoln, NE 68588-6205, USA; enolasco2@huskers.unl.edu (E.N.); philip.johnson@unl.edu (P.E.J.)
[2] Proteomics and Metabolomics Facility, Nebraska Center for Biotechnology, University of Nebraska-Lincoln, Lincoln, NE 68588-0665, USA; mnaldrett@unl.edu (M.N.); salvarez@unl.edu (S.A.)
* Correspondence: kaustav.majumder@unl.edu

Table of content

1.	**Figure S1.** White Leghorn (WLH) and Rhode Island Red (RIR) whole hydrolysate concentration optimization for ROS DCFH-DA assay in Caco-2 cells.	ii
2.	**Figure S2.** MS2 spectra for matched peptides ID described in Table 1. The parent ion accuracy is included for each peptide ID. Additional data for all peptides from the samples is given in Supp. Data 2.	

Figure

Figure S1. White Leghorn (WLH) and Rhode Island Red (RIR) <3000 Da peptide fraction concentration optimization for ROS DCFH-DA assay in Caco-2 cells. Area under the curve (A, B) and cellular antioxidant activity (C, D) for White Leghorn (A, C) and Rhode Island Red (B, D). Concentrations are expressed in µg/mL. Ind.: Inducer control (600 µM AAPH + 25 µM DCFH-DA), Inh.: Inhibitor control (10 µM quercetin +600 µM AAPH + 25 µM DCFH-DA), n=4.

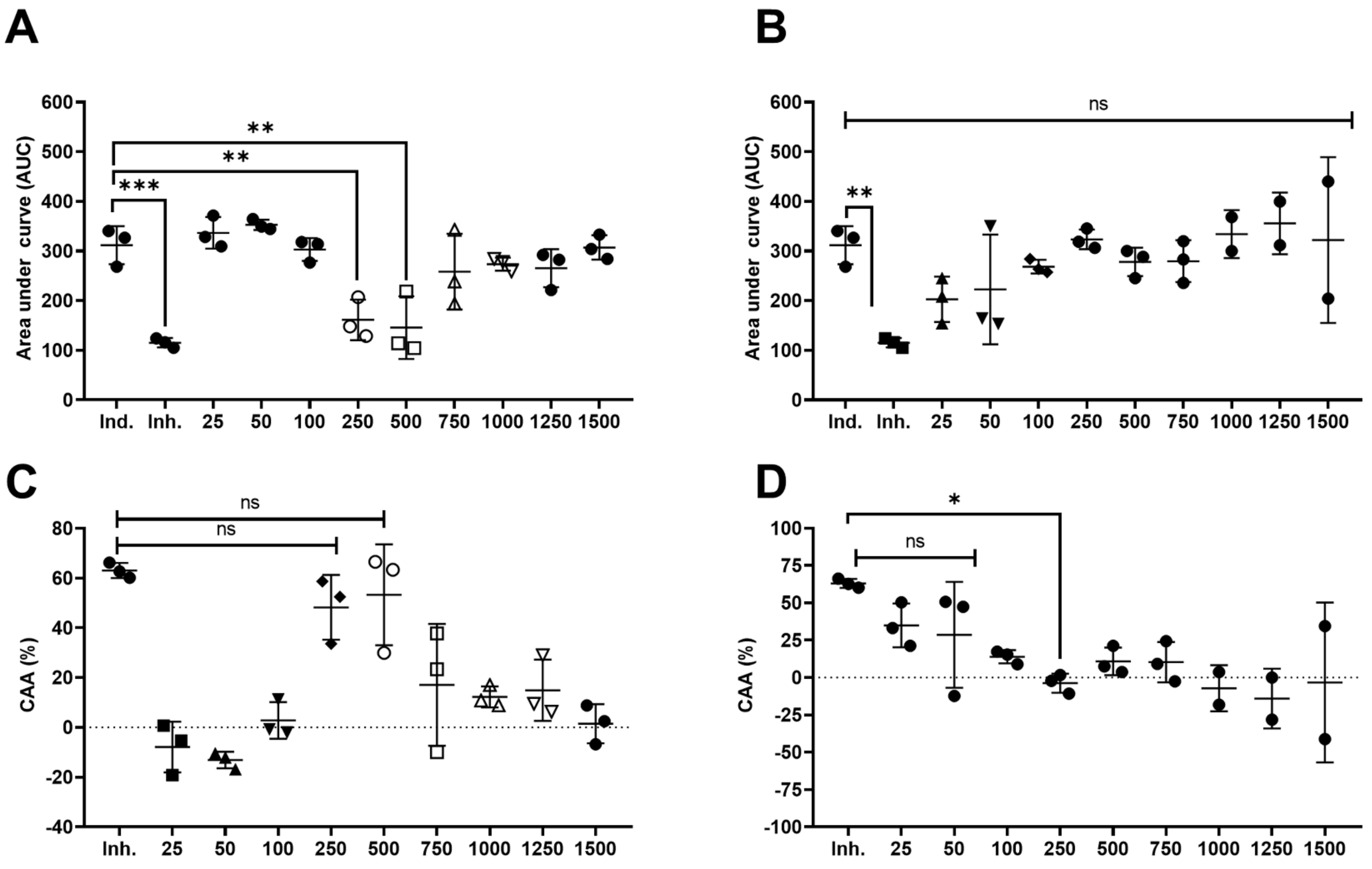

Figure S2. MS2 spectra for matched peptides ID described in Table 1. The parent ion accuracy is included for each peptide ID. Additional data for all peptides from the samples is given in Supp. Data 2.

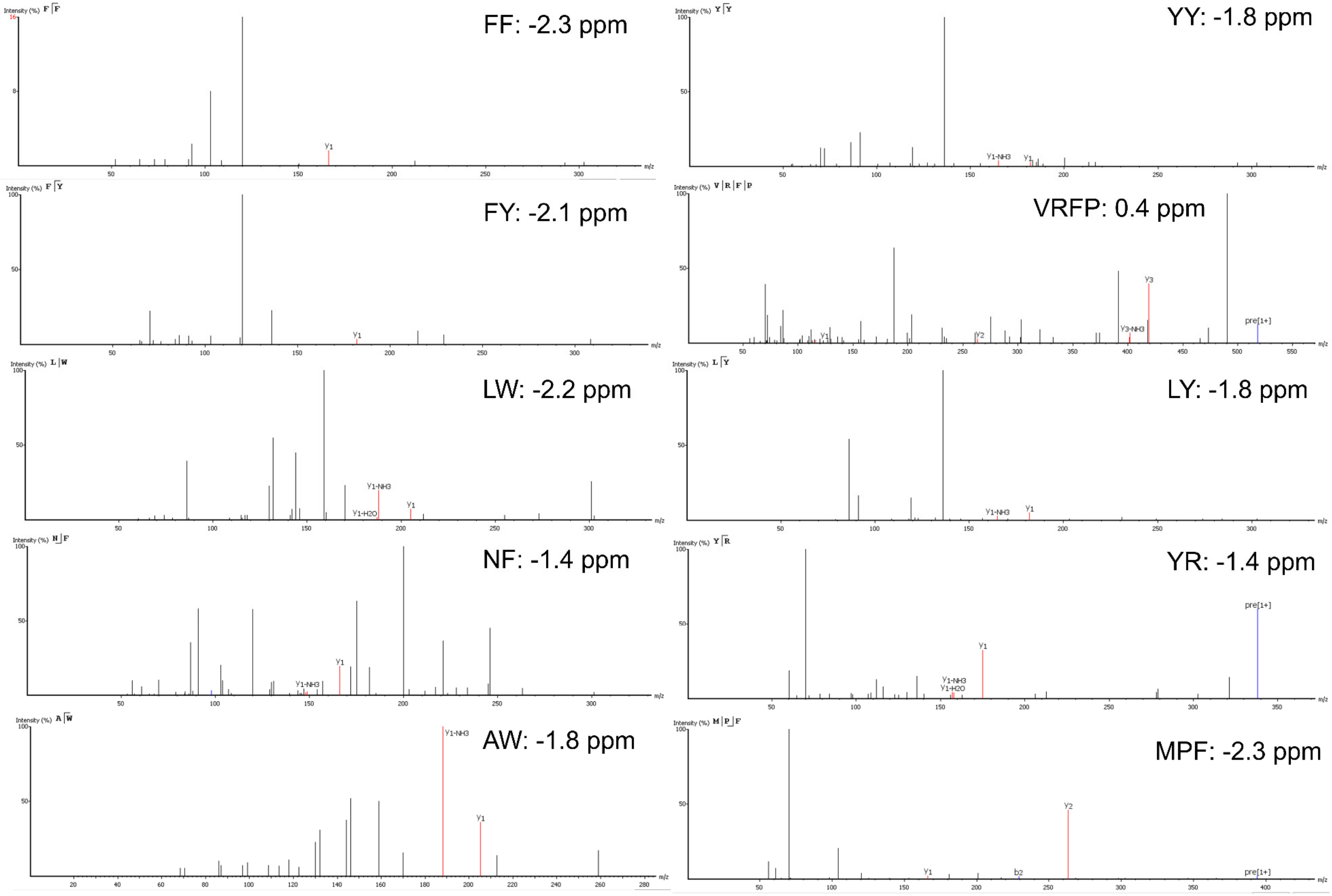

Supplement: Supplementary file 1 [file nutrients-13-04232-s001.zip › Supplementary Data1.pdf]
